# Whose Mandate? Reconsidering the Role of Patients and Health Consumers in Defining the Role of Nurses

**DOI:** 10.1111/inr.70186

**Published:** 2026-05-19

**Authors:** Faustino Jerome Babate, Nuzul Qur'aniati

**Affiliations:** ^1^ Department of Pediatric and Maternity Nursing Universitas Airlangga Surabaya Indonesia; ^2^ Philippine Christian University Manila Philippines; ^3^ Filipino Nursing Diaspora Network Sydney Australia

The recent article by Köhler et al. (2026) on the role and mandate of nurses in disasters represents a timely and valuable contribution to the growing body of scholarship addressing the strategic position of nursing within health system resilience and disaster governance. By employing a multi‐stage Delphi process to generate expert consensus across Germany, Austria, and Switzerland, the authors highlight the urgent need to clarify and codify nurses’ professional mandate in disaster preparedness, response, and recovery. The study appropriately emphasizes the importance of strengthening nursing leadership, educational preparedness, and policy recognition within national disaster management frameworks.

While these contributions are significant and highly relevant for nursing policy development, the article also draws attention—perhaps unintentionally—to an enduring methodological and policy challenge within nursing research: the limited involvement of patients and health consumers in consensus‐based studies. From the perspective of patients and health consumers, the absence of such voices raises important questions about whose perspectives ultimately shape the understanding of nursing roles within health systems.

The Delphi panel convened by Köhler et al. (2026) included experts from nursing, disaster management, health policy, and related sectors.

However, patients, community representatives, and health consumers were not included among the expert participants. While this approach is consistent with many traditional Delphi studies, it reflects a broader pattern in health policy research where professional expertise is privileged over experiential knowledge. Yet contemporary health system governance increasingly recognizes that patients and communities possess unique insights into care delivery, system responsiveness, and the social consequences of health policy decisions (WHO, 2024).

Within the scope of *INR*, which frequently addresses global nursing leadership, policy, and health system reform, the question of patient and public involvement becomes particularly relevant. Nursing is widely recognized as a patient‐centered profession (Davoodvand et al. 2016), grounded in advocacy, trust, and relational care. When studies seek to define the societal mandate of nurses—especially in high‐stakes contexts such as disasters—it is reasonable to ask whether those who receive care should also help define that mandate.

Evidence from recent health policy research suggests that participatory approaches can substantially enrich consensus‐building processes. Studies examining patient and public involvement in research priority setting demonstrate that patient inclusion often shifts the focus of deliberations toward issues such as communication, accessibility, equity, and continuity of care (Boaz et al. 2018; Ayton et al. 2021). These dimensions are particularly relevant in disaster contexts, where vulnerable populations frequently face barriers to accessing timely, coordinated healthcare services.

Similarly, participatory governance frameworks have emphasized the importance of community engagement in strengthening health system resilience. In disaster preparedness and response, community perspectives can illuminate how health systems function under extreme conditions and how frontline professionals—including nurses—are perceived by the populations they serve (Abimbola et al. 2014). Integrating such perspectives may provide a more comprehensive understanding of the expectations placed upon nurses during emergencies, crises, and disasters.

The development of national nursing research agendas provides a useful point of comparison. In several countries, priority‐setting initiatives have begun to incorporate patients and caregivers alongside clinicians, policymakers, and academics. These initiatives recognize that experiential knowledge complements professional expertise and can help ensure that research priorities align with the real‐world needs of patients and communities (Manafò et al. 2018). When the objective is to define the societal role and mandate of nursing, such participatory approaches may be particularly valuable.

There are also important policy implications when patient and consumer perspectives are absent from such deliberations. First, policies designed without patient input risk overlooking aspects of care that matter most to communities, including trust, cultural responsiveness, and communication during crises. Disasters often expose existing inequalities within health systems, and patients and caregivers are frequently the first to experience these gaps.

Second, excluding patient voices may reinforce technocratic approaches to health policy development. Disaster governance typically involves complex institutional structures dominated by professional and administrative actors. While technical expertise is essential, disaster response also involves ethical decisions concerning triage, vulnerability, and resource allocation. Patient and community perspectives can help contextualize these ethical considerations and contribute to more socially legitimate policy frameworks.

Third, including patients and health consumers may strengthen public trust in the nursing profession and the institutions that govern healthcare systems. Trust has emerged as a critical component of effective disaster response and health system resilience. Engaging communities in defining nursing roles may reinforce the perception of nursing not only as a clinical profession but also as a trusted partner in safeguarding public health.

For nursing policy research published in INR, this raises an important methodological consideration. Future consensus studies examining the role of nurses—whether in disaster preparedness, public health, or broader health system governance—could benefit from incorporating structured patient and community participation. Methodological innovations such as participatory Delphi panels, mixed‐stakeholder advisory groups, or citizen‐engagement processes may help bridge the gap between professional expertise and lived experience.

Importantly, incorporating patient and consumer voices should not be interpreted as diminishing the authority of nursing expertise. Rather, it reflects an evolution toward collaborative knowledge production in health policy research. Nurses bring clinical expertise, ethical responsibility, and system‐level insight, while patients and communities contribute lived experiences of vulnerability, resilience, and care relationships. Together, these perspectives can generate more comprehensive and socially responsive understandings of nursing roles.

The work by Köhler et al. (2026) provides an important foundation for advancing discussions on nurses’ mandate in disasters and offers valuable insights for nursing policy in Europe and beyond.

As this policy conversation continues within the international nursing community, integrating patient and health consumer perspectives may further strengthen the relevance, legitimacy, and societal impact of such efforts.

Ultimately, defining the role and mandate of nurses is not solely a professional exercise; it is a societal endeavor. Including the voices of patients and communities—those whom nurses ultimately serve—may help ensure that this mandate reflects not only professional aspirations but also the needs, expectations, and experiences of the populations whose health systems seek to protect.

## Funding

LPDP on behalf of the Ministry of Higher Education, Science, and Technology and managed under EQUITY Contract No. ‘4300/B3/DT.03.08/2025 and 297/UN3/HK.07.00/2025’
